# Small RNA sequencing provides candidate miRNA-target pairs for revealing the mechanism of apomixis in *Zanthoxylum bungeanum*

**DOI:** 10.1186/s12870-021-02935-5

**Published:** 2021-04-13

**Authors:** Xitong Fei, Yu Lei, Yichen Qi, Shujie Wang, Haichao Hu, Anzhi Wei

**Affiliations:** 1 College of Forestry, Northwest Agriculture and Forestry University, Xianyang, 712100 China; 2 Research Centre for Engineering and Technology of Zanthoxylum State Forestry Administration, Yangling, Xianyang, 712100 China

**Keywords:** Apomixis, *AGAMOUS*, miRNA-target pairs, miR172, *TOE3*, *Zanthoxylum bungeanum*

## Abstract

**Background:**

Apomixis is a form of asexual reproduction that produces offspring without the need for combining male and female gametes, and the offspring have the same genetic makeup as the mother. Therefore, apomixis technology has great application potential in plant breeding. To identify the apomixis types and critical period, embryonic development at different flower development stages of *Zanthoxylum bungeanum* was observed by cytology.

**Results:**

The results show that the S3 stage is the critical period of apomixis, during which the nucellar cells develop into an adventitious primordial embryo. Cytological observations showed that the type of apomixis in *Z. bungeanum* is sporophytic apomixis. Furthermore, miRNA sequencing, miRNA-target gene interaction, dual luciferase reporter assay, and RT-qPCR verification were used to reveal the dynamic regulation of miRNA-target pairs in *Z. bungeanum* apomixis. The miRNA sequencing identified 96 mature miRNAs, of which 40 were known and 56 were novel. Additionally, 29 differentially expressed miRNAs were screened according to the miRNAs expression levels at the different developmental stages. Kyoto Encyclopedia of Genes and Genomes (KEGG) and Gene Ontology (GO) enrichment analyses showed that the target genes of the differentially expressed miRNAs were mainly enriched in plant hormone signal transduction, RNA biosynthetic process, and response to hormone pathways.

**Conclusions:**

During the critical period of apomictic embryonic development, miR172c significantly reduces the expression levels of *TOE3* and *APETALA 2* (*AP2*) genes, thereby upregulating the expression of the *AGAMOUS* gene. A molecular regulation model of miRNA-target pairs was constructed based on their interactions and expression patterns to further understand the role of miRNA-target pairs in apomixis. Our data suggest that miR172c may regulates *AGAMOUS* expression by inhibiting *TOE3* in the critical period of apomixis.

**Supplementary Information:**

The online version contains supplementary material available at 10.1186/s12870-021-02935-5.

## Background

The most common form of reproduction in flowering plants is double fertilization. Here, the male and female gametophytes combine to form a new plant [1]. However, another mode of plant reproduction can produce offspring without sexual reproduction, known as apomixis. This is a reproductive mode that directly produces offspring, without combining the male and female gametes. The genetic makeup of the offspring in apomictic species is completely consistent with that of the female parent, and so retains any superior traits the female parent might possess. Hence, apomixis has considerable application potential in crop genetic breeding.

There are three types of apomixis, depending on the source of the embryo: diplospory, apospory, and sporophyte apomixis. Diplospory is when an egg cell in the unreduced diploid embryo sac develops into an embryo [2]. This type can be found in plants such as fleabanes [3]. Apospory is the reproductive process in which the nucellus cells form an unreduced embryo sac through mitosis, and the initial cells develop into the embryo. This type occurs in plants such as bahiagrass [4]. Both these forms of reproduction belong to gametophytic apomixis. Sporophyte apomixis is a process in which an adventitious polyembryony is formed by the mitotic division of nucleated cells. This type can be found in plants such as *Cyrtogonellum caducum* [5–7]. In such cases, the endosperm may not form autonomously by fertilization during the process of apomixis, and pollen and polar nuclei might be required to form a triploid endosperm through pseudogamy [8, 9].

Recent research on the mechanisms of apomixis has made unprecedented progress, and various regulatory factors have been shown to be involved in the apomictic process. *Gibberellin-insensitive dwarf protein 1* (*BbGID1*) is specifically expressed in the nucellus of *Brachiaria brizantha*. Overexpression of *AtGID1* results in the differentiation of *Arabidopsis thaliana* megaspore mother cell-like cells [10]. *Baby boom 1* (*BBM1*) is a member of the AP2 family of transcription factors expressed in rice sperm cells. Its ectopic expression in egg cells can cause the rice to reproduce apomictically, without fertilization [11]. In addition, a long non-coding RNA (lncRNA), PN_LNC_N13, has differential expression in sexually reproducing and apomictic *Paspalum notatum*, indicating that lncRNAs might be involved in apomixis regulation [12]. Furthermore, small RNA (sRNA) is an indispensable regulator in apomixis. Argonaute 9-dependent sRNA silencing plays a key role in the development of *Arabidopsis* ovules, and the sRNA-dependent silencing of plant gametes is required for epigenetic reprogramming of the accompanying cells [13]. In the ovules of apomictic citrus, the expression level of miRN23-5P in monoembryonic varieties was reported to be significantly higher than in polyembryonic varieties, and its expression is negatively correlated with the expression of its regulated target genes [14]. In *Eragrostis curvula*, miRNA-mRNA interaction analysis showed that miRNA might participate in apomixis by regulating a MADS-box transcription factor gene and a transposon [15]. Therefore, expression analysis of miRNA-target genes would likely be a valuable tool for elucidating the apomictic regulatory networks. Although some progress has been made in the study of apomixis, its full regulatory network remains obscure and incomplete.

*Zanthoxylum bungeanum* (ZB) is a species of the genus *Zanthoxylum*, which is widely distributed in Shaanxi, Gansu, Sichuan, Shandong, and Hebei Provinces, and many other places around China [16]. The skin of the ZB fruit is a well-known traditional Chinese seasoning herb. It is widely used in cooking because of its unique numbing properties. It is also used in traditional Chinese medicine to treat various diseases [17]. ZB has apomictic characteristics, and so it is a suitable model in which to study apomixis. In this study, cytological observations, transcriptomics, dual-luciferase reporter assay, RT-qPCR, and miRNA-target gene interaction verification were used to study the molecular mechanism of ZB apomixis. Our results provide references for the study of the apomixis developmental process and mechanism.

## Results

### Identification of Apomixis in *Zanthoxylum bungeanum*

**Fig. 1:**
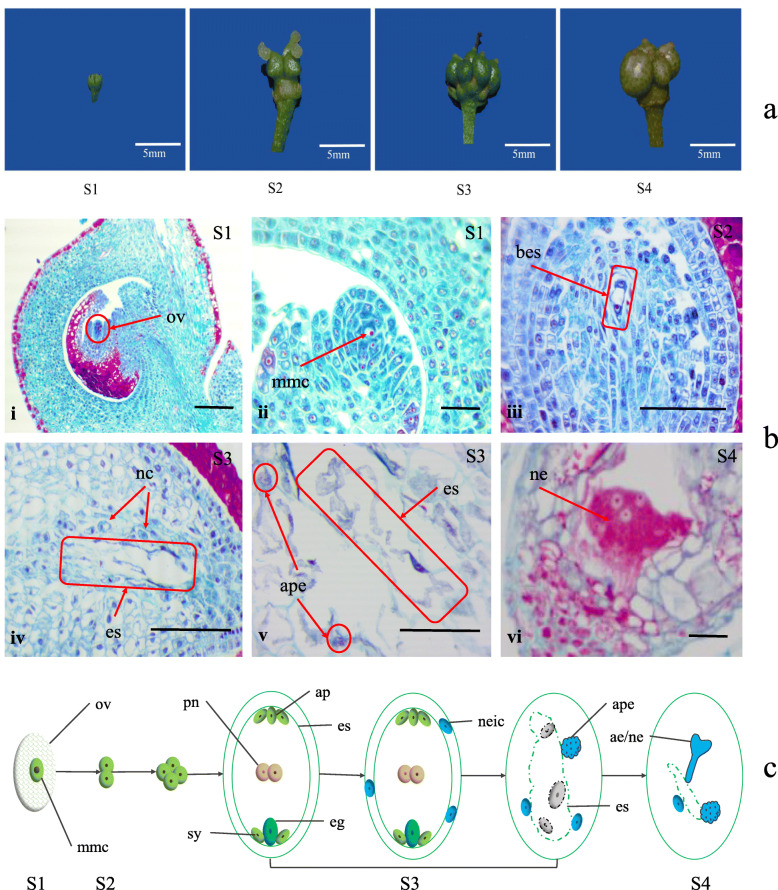
Cytological observations of apomixis in *Zanthoxylum bungeanum.* **a** Different developmental stages of the *Zanthoxylum bungeanum* fruit. **b** Cytological observation of apomixis in *Zanthoxylum bungeanum*. (i-ii) The megaspore mother cell is formed in the S1. (iii) The binuclear embryo sac is formed in the S2. (iv-v) The embryo sac decomposes, and the nucellus cells develop into the adventitious primordial embryo in the S3. (vi) The adventitious primordial embryo develops into a nucellus embryo in the S4. Scale bars = 20 μm. **c** A model diagram of adventitious embryogenesis in *Zanthoxylum bungeanum.* ae: adventitious embryo, ap: antipodals, ape: adventitious primordial embryo, bes: binuclear embryo sac, eg: egg, es: embryo sac, mmc: megaspore mother cell, nc: nucellar cell, ne: nucellus embryo, neic: nucellar embryo initial cells, ov: ovule, pn: polar nuclei, sy: synergids

The critical period of apomixis embryo development needs to be identified when studying the mechanism of apomixis. To do that, we performed cytological observations on the collected flowers at different developmental stages (Fig. 1a). The results showed that the ovule appears before flowering (S1) and contains a megaspore mother cell (Figs. 1b i and ii). In S2, the megaspore mother cell in the ovule divides to form a binuclear embryo sac (Fig. 1b iii). In S3, the binuclear embryo sac develops into a mature embryo sac, but the embryo sac decomposes and cannot function normally (Fig. 1b iv). Near the embryo sac, the nucellus cells continue to differentiate to form the adventitious primordial embryo (Fig. 1b v). Finally, the adventitious primordial embryo develops into a nucellus embryo in S4 (Fig. 1b vi). A model diagram of adventitious embryogenesis was established to show apomixis in ZB. Based on this diagram, ZB was found to have the characteristics of sporophytic apomixis. The differentiation of nucellar cells into the adventitious primordial embryo at the S3 stage is the most critical period for apomixis.

### Small RNA sequencing profile

Small RNA sequencing was performed by the Illumina HiSeq 2500 sequencing platform (Illumina, San Diego, CA, USA) on 12 ZB samples at the four apomixis developmental stages. Sequencing obtained 62,810,130 reads and 60,347,610 clean reads, among which high-quality reads accounted for more than 99.95% (Table 1). The Bowtie software was used to analyze the location and distribution of miRNAs on the reference sequence. The analysis results showed that more than 62% of the small RNAs could be located on the reference sequence (*Zanthoxylum bungeanum* non-reference genome transcriptome spliced transcript as reference sequence). The number of reads mapped to the same strand in the reference sequence exceeded 50%, while the number of reads mapped to the opposite strand of the reference sequence comprised less than 10%.

**Table 1:** Summary of small RNA sequencing results

Sample	S1	S2	S3	S4
Total reads	19,572,690 (100%)	14,258,144 (100%)	13,540,799 (100%)	15,438,497 (100%)
N% > 10%	491 (0.00%)	223 (0.00%)	22 (0.00%)	41 (0.00%)
Low quality	8922 (0.05%)	5375 (0.04%)	3203 (0.02%)	4338 (0.03%)
5′ adapter contamination	24,207 (0.12%)	18,667 (0.13%)	13,689 (0.10%)	14,148 (0.09%)
3′ adapter null or insert null	943,999 (4.82%)	740,543 (5.19%)	263,635 (1.95%)	320,439 (2.08%)
With ploy A/T/G/C	41,579 (0.21%)	10,186 (0.07%)	38,713 (0.29%)	10,100 (0.07%)
Clean reads	18,553,492 (94.79%)	13,483,150 (94.56%)	13,221,537 (97.64%)	15,089,431 (97.74%)
Total sRNA	10,436,878 (100%)	8,007,808 (100%)	10,348,535 (100%)	11,058,825 (100%)
Mapped sRNA	8,109,791 (77.70%)	7,269,789 (90.78%)	6,456,441 (62.39%)	10,301,776 (93.15%)
“+” Mapped sRNA	7,381,437 (70.72%)	6,504,225 (81.22%)	5,689,563 (54.98%)	9,825,133 (88.84%)
“-” Mapped sRNA	728,354 (6.98%)	765,564 (9.56%)	766,878 (7.41%)	476,643 (4.31%)

(1) Sample: sample id. (2) Total reads: count of original sequence data. (3) N% > 10%: The number of reads with N content exceeding 10% and the proportion of the total raw reads number. (4) Low quality: the number of low-quality reads. (5) 5′ adapter contamination: The number of reads in the 5′ adaptor. (6) 3′ adapter null or insert null: the number of reads without 3′ adapter or insert. (7) With ploy A/T/G/C: the number of reads containing ploy A/T/G/C. (8) Clean reads: The number of clean reads finally obtained. (9) Total sRNA: The total number of reads obtained in each sample. (10) Mapped sRNA: The number of reads mapped to the reference sequence. (11) “+” Mapped sRNA: The number of reads mapped to the same strand in the reference sequence direction. (12) “-” Mapped sRNA: The number of reads mapped to the opposite strand of the reference sequence

**Table 2:** miRNAs and their target genes

miRNA	Mature miRNA sequence	Target gene description	Target gene ID
ath-miR172c	AGAAUCUUGAUGAUGCUGCAG	*Transcription factor TOE3*	XM_006485885.2
ath-miR172c	AGAAUCUUGAUGAUGCUGCAG	*APETALA 2*	XM_006494404.2
ath-miR167d	UGAAGCUGCCAGCAUGAUCUGG	*Alpha,alpha-trehalose-phosphate synthase*	XP_006432796.1
ath-miR319a	UUGGACUGAAGGGAGCUCCCU	*Transcription factor GAMYB*	XM_006471200.2
ath-miR160a-5p	UGCCUGGCUCCCUGUAUGCCA	*Auxin response factor 1*	XM_006485635.2
ath-miR858a	UUUCGUUGUCUGUUCGACCUU	*Transcription factor MYB3*	XP_006474484.1
ath-miR166a-5p	GGACUGUUGUCUGGCUCGAGG	*Abscisic acid-insensitive 5-like protein 7*	KDO59710.1
ath-miR162a-3p	UCGAUAAACCUCUGCAUCCAG	*Endoribonuclease Dicer homolog 1*	XP_006444699.1
ath-miR157d	UGACAGAAGAUAGAGAGCAC	*Squamosa promoter-binding-like protein*	XP_006477710.1
ath-miR156b-3p	UGCUCACCUCUCUUUCUGUCAGU	*DEAD-box ATP-dependent RNA helicase*	XR_370496.2
ath-miR5653	UGGGUUGAGUUGAGUUGAGUUGGC	*Auxin-responsive protein IAA26*	XP_006465700.1
ath-miR319a	UUGGACUGAAGGGAGCUCCCU	*Transcription factor TCP2*	XP_006449018.1

**Fig. 2:**
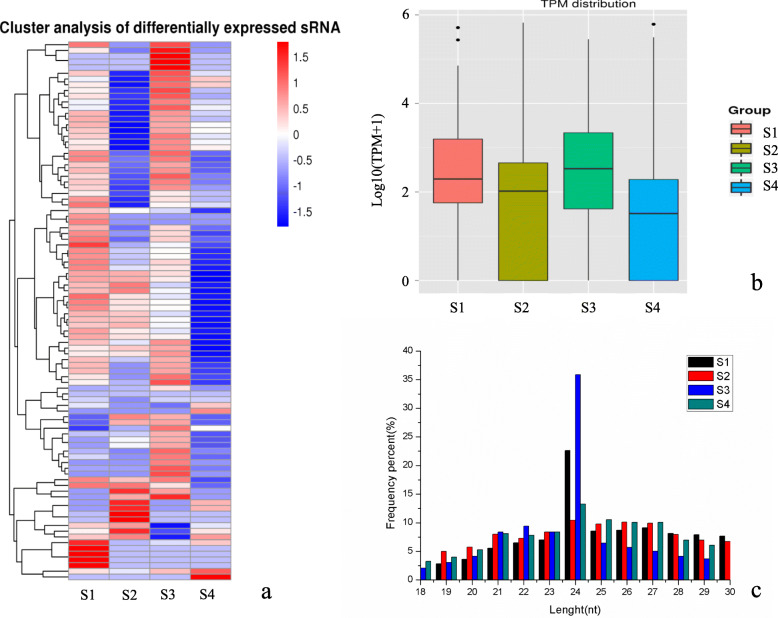
The expression pattern of miRNAs in *Zanthoxylum bungeanum* apomictic fruit developmental stages. **a** Cluster analysis of differentially expressed sRNA. **b** Length distribution of transcripts per million reads (TPM). **c** Length distribution of the miRNAs

A total of 96 mature miRNAs were identified, of which 40 were known, and 56 were novel. A total of 106 miRNA hairpins were also identified, 49 known and 57 novel (Table S1). Transcripts per million reads (TPM) can represent the miRNAs expression level. According to the log10 (TPM + 1) value of miRNAs, the differentially expressed miRNAs in the different groups were clustered to determine their expression patterns at the four ZB fruit developmental stages (Fig. 2a). The miRNAs expression pattern was directly related to their functions. We primarily analyzed the differentially expressed miRNAs during the critical apomictic embryogenesis period (S3) to study the relationship between miRNAs and apomixis. Additionally, large differences in the TPM distribution of miRNAs were noted between the four ZB fruit developmental stages (Fig. 2b). The length range of animal sRNA is 18–35 nt, while the length range of plant sRNA is 18 ~ 30 nt. The length distribution peak can determine the type of sRNA. For example, the length of miRNA is concentrated in the 21–22 nt range, while the siRNA length is mostly of 24 nt. The miRNAs in the four ZB fruit development stages were 18–30 nt long, with miRNA of 24 nt being the most abundant in all stages (Fig. 2c). The differences in the length distribution of ZB miRNAs offer indirect evidence that there are different regulatory mechanisms at different apomixis stages.

### Differentially expressed miRNA analysis in Apomixis

**Fig. 3:**
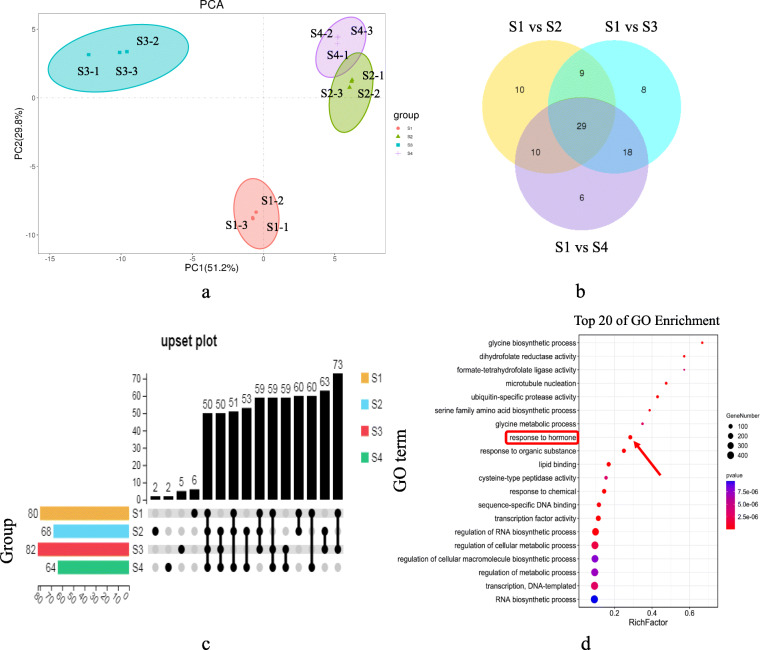
Functional enrichment analysis of differentially expressed miRNAs and their target genes at the four *Zanthoxylum bungeanum* fruit developmental stages. **a** Principal component analysis of fruits at the four developmental stages. **b** Venn diagram of differentially expressed miRNAs at the different developmental stages. **c** Quantitative distribution of mature miRNAs at the different developmental stages. The bottom panel shows the number distribution of miRNAs at different developmental stages, and the connected black dots represent the number of miRNAs shared by different samples. **d** Gene Ontology (GO) enrichment analysis of the differentially expressed miRNAs target genes

Principal component analysis (PCA) was performed on 12 ZB samples from the four fruit developmental stages According to the miRNAs expression level or TPM. The results showed that PC1 and PC2 explained together 81.0% of the sample differences, and sample clustering into groups showed that they had good repeatability and met the data analysis requirements (Fig. 3a). Analysis of the differentially expressed miRNAs in the experimental groups can screen for the miRNAs that play key roles in the process. Therefore, we used the DESeq software to screen and analyze the miRNAs at the four ZB apomixis stages. We found 29 differentially expressed miRNAs (Fig. 3b). Of these, 15 were known miRNAs, and 14 were novel. An Upset plot was drawn based on the number distribution of 96 mature miRNAs at the different developmental stages (Fig. 3c). The results showed that the number of mature miRNAs detected in S3 was the largest (82), while that in S4 was the smallest (64). Furthermore, the numbers of miRNAs unique to developmental stages S1, S2, S3, and S4 were 6, 2, 5, and 2, respectively. The number of miRNAs shared by the four developmental stages was 50.

miRNA participates in various life processes by regulating the expression of target genes or inhibiting their translation. Therefore, it is necessary to determine the miRNA functions through their interactions with target genes and their expression levels. We used the Kyoto Encyclopedia of Genes and Genomes (KEGG) to analyze pathways enrichment of differentially expressed genes during apomixis in ZB. The results showed that many differentially expressed genes were associated with RNA transport, protein synthesis on the plant hormone signal transduction (ko04075), RNA transport (ko03013), and steroid biosynthesis (ko00100; Figure S1). Many substances were also synthesized, including fatty acids, starch, sucrose, and folate. In addition, Gene Ontology (GO) enrichment analysis was performed on the target genes of differentially expressed miRNAs (Fig. 3d). The results showed that many differentially expressed genes were enriched in several pathways, including RNA biosynthetic process (GO:0032774), transcription factor activity (GO:0003700), response to hormone (GO:0009725), and more. Among them, 457, 167, and 81 differentially expressed genes were enriched in the RNA biosynthetic process, transcription factor activity, and response to hormone pathways, respectively, indicating that scores of RNA and transcription factors were synthesized, activating the hormone response pathways during ZB apomixis. KEGG and GO enrichment analyses of the differentially expressed miRNAs target genes showed that many genes were enriched in pathways related to hormone synthesis and regulation, indicating hormones that might be involved in ZB apomixis regulation.

### Interaction analysis of the miRNAs and their target genes

**Fig. 4:**
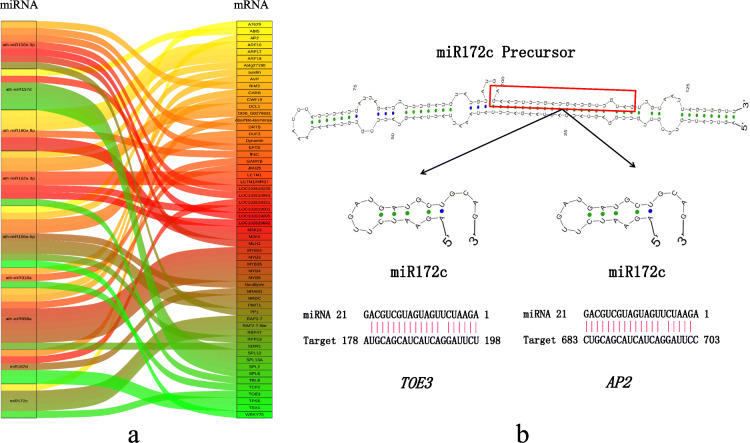
miRNAs and their target genes. **a** Sankey diagram of miRNAs and their target genes. **b** miR172c precursor, mature body, and binding site with target genes

**Fig. 5:**
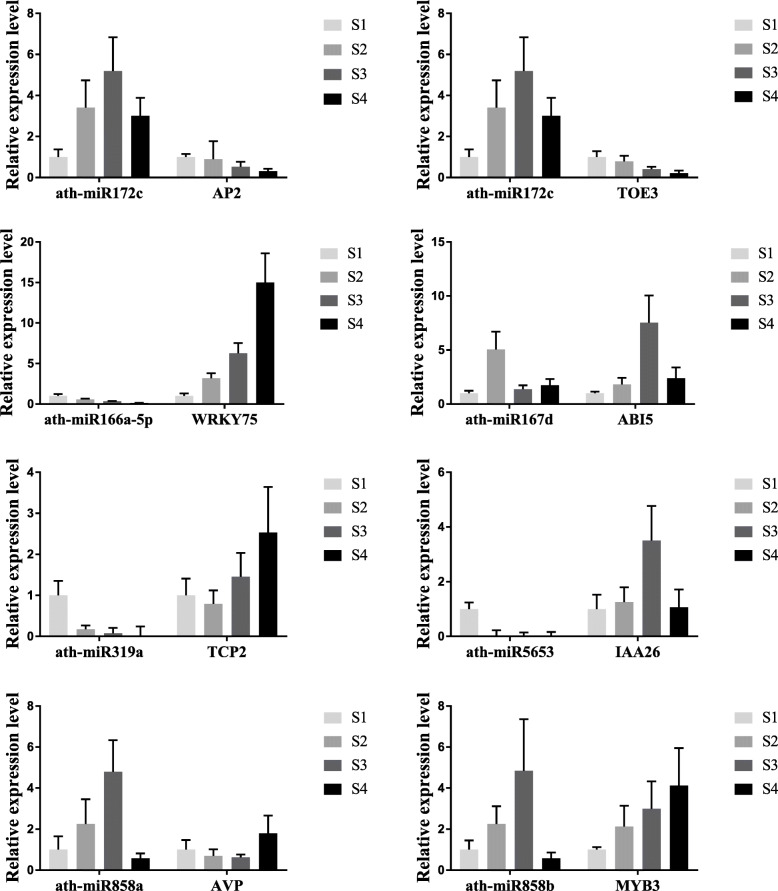
Relative expression levels of miRNAs and their target genes involved in apomixis by RT-qPCR

A comprehensive analysis of miRNA and mRNA expression profiles helped identify functional miRNA-mRNA interaction pairs involved in the apomictic processes (Fig. 4a and Table 2). Eight miRNAs in the four developmental stages were significantly differentially expressed. These were detected by quantitative reverse transcription PCR (RT-qPCR) to verify the validity of the sequencing results (Fig. 5). The results showed that the miRNAs sequencing and RT-qPCR results were essentially consistent, confirming their reliability. Additionally, we predicted the target genes of differentially expressed miRNAs, and the results showed that they were involved in genes and regulatory factors of multiple life processes, including hormone-related *ABI5*, *IAA26*, and *TOE3*, and several transcription factors such as *WRKY75*, *MYB3,* and *TCP2*. Among them, miR172c had two target genes, *TOE*3 and *AP2*. The mature miR172c can form complementary pairs with both target genes (Fig. 4b). It had a high expression level in the key ZB apomixis stage (S3), implying that it and its target genes played an important role in apomixis.

The relative expression levels of miRNAs and their target genes were analyzed by RT-qPCR to further determine the interactions between them (Fig. 5). The results showed that during ZB fruit development, the expression levels of the eight differentially expressed miRNAs and their target genes were negatively correlated, preliminarily verifying the mode of interaction between them.

### Verification of the interaction between miRNA and its target genes

**Fig. 6:**
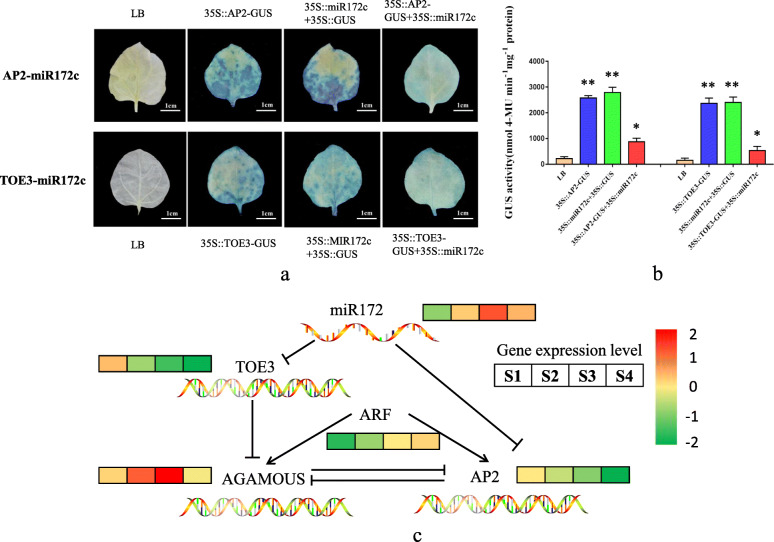
Verification of the interactions between miR172c and its target genes. **a** GUS immunohistochemistry. **b** GUS activity. **c** The miRNA-target interactions, and their expression patterns during apomixis

We constructed miRNA and target gene sequences on pCA1301 and pBI121 vectors, respectively, to demonstrate the interactions between them. These were transferred to tobacco leaves, and the expression of the GUS protein was evaluated to determine whether there was an interaction between the miRNA and its target gene [18]. The 35S::*AP2*-GUS, 35S::*TOE3*-GUS, and 35S::miR172c-GUS produced a large amount of GUS protein after injection into tobacco leaves, while 35S::*AP2*-GUS + 35S::miR172c-GUS and 35S::*TOE3*-GUS + 35S::miR172c-GUS significantly reduced GUS protein activity (Fig. 6a and b). These results suggest that miR172c could cleave *TOE3* and *APETALA 2* (*AP2*) genes and thus block the synthesis of the GUS protein, confirming that *TOE3* and *AP2* were its target genes.

A molecular regulation model was constructed based on miR172c-*AP2* and miR172c-*TOE*3 interactions to understand the molecular process in ZB apomixis (Fig. 6c). *AGAMOUS* belongs to the MADS-Box transcription factor class C genes, and *AP2* belongs to the MADS-Box transcription factor class A genes. Class A and C genes have antagonistic effects on each other. *AGAMOUS* can control the development of stamens and carpels and regulate the activity of floral meristems, playing an important role in apomixis [19, 20]. *AGAMOU*S expression level gradually increased during fruit development, especially during the critical period of ZB apomictic embryogenesis (S3). The expression level of *AP2*, on the other hand, gradually decreased during development. The miRNA-target genes interaction experiment showed that miR172c could cleave *TOE*3, and TOE3 could inhibit *AGAMOUS* expression (Fig. 6c). Therefore, miR172c can increase *AGAMOUS* expression abundance by inhibiting *TOE3* transcription level during ZB apomixis. Besides, during fruit development, the expression level of the auxin response factor (*ARF*) increased gradually, suggesting that hormones might play an important role in apomixis.

## Discussion

**Fig. 7:**
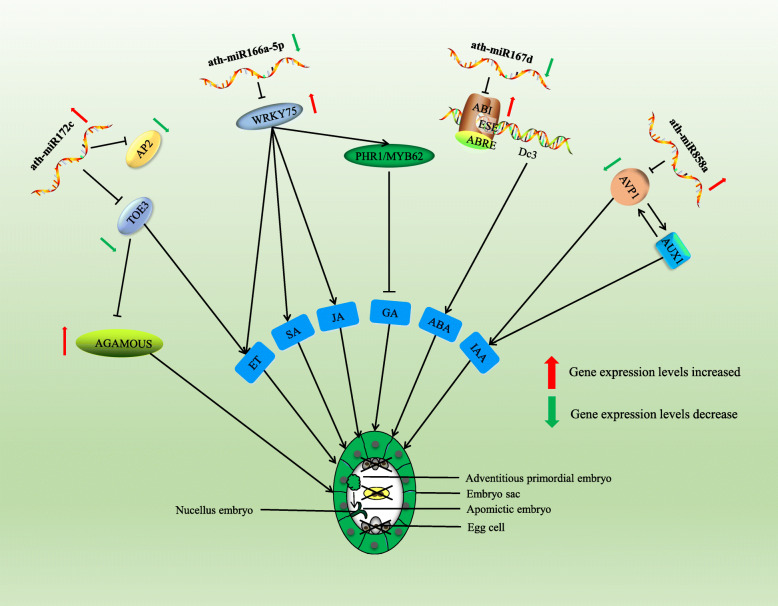
miRNA-targets phytohormone regulation model during apomixis in *Zanthoxylum bungeanum*

Apomixis results in the offspring inheriting the complete genotype of the female parent, thus having broad application potential in agricultural heterosis fixation. However, the mechanism of apomixis is still unclear. Clarifying the apomixis embryogenesis process is a prerequisite for studying its mechanism. We analyzed the apomixis embryogenesis process in ZB by cytological observations, concluding that ZB was the sporophytic apomixis type and that S3 was a critical period of apomixis. In this study, miRNA sequencing, miRNA-target gene interactions, dual luciferase reporter assay, and RT-qPCR verification were used to reveal the dynamic regulation of miRNA-target pairs in ZB apomixis. The miRNAs are involved in various biological processes and are among the most important factor groups regulating organisms [21–23]. Many differentially expressed miRNAs were detected in ZB apomixis, indicating that they probably play an important role in the process. Studying their interactions with their target genes during apomixis could identify candidate genes and new ideas to help elucidate the apomixis mechanism. Using miRNA sequencing, miRNA-target gene interaction experiments, and RT-qPCR verification, we showed that miR172 inhibits the activity of *TOE3* and *AP2* during S3, thereby increasing the expression level of *AGAMOUS*. Because of that, miR172 might be a key regulator of apomixis (Fig. 7). Moreover, miR172 is evolutionarily conserved among angiosperms and is widely involved in plant floral organ development. It controls the flowering time of floral organs by regulating multiple MADS-box transcription factors [24, 25].

The functional analysis of differentially expressed miRNAs and their target genes in apomixis showed that many target genes play an active role in hormone synthesis and regulation. To further understand the possible role of miRNAs in apomixis, based on the analysis results and previous studies, a miRNA interaction regulation model was established. Moreover, numerous studies have shown that hormones can induce plant somatic cells to form embryos and activate plant cell totipotency [26, 27]. Studies have also detected many hormonal changes during seed embryo development [28, 29]. In *Brassica napus*, jasmonic acid is a tissue-specific hormone involved in gene and protein expression during embryonic development. Abscisic acid (ABA) has been shown to induce cell differentiation during somatic embryogenesis in *Cunninghamia lanceolate*, thus promoting embryonal mass growth [30]. The auxin-responsive gene, ABA-related gene, and gibberellin-related gene are all regulated by miR393. This miRNA plays an important role in barley hormone synthesis and embryonic development [31]. Based on our analysis, it is inferred that miRNAs regulate the hormone synthesis by interacting with their target genes, thereby activating the totipotency of plant cells that participate in apomixis.

*WRKY75* is a target gene of ath-miR166a-5p, which binds and degrades it to block its transcription and inhibit its function. RT-qPCR showed that *WRKY75* and ath-miR166a-5p expression trends during ZB apomixis were negatively correlated, and that *WRKY75* relative expression level was significantly increased. *WRKY75* is a transcription factor that interacts with many hormones [32]. AtWRKY75 can activate the pathways of jasmonic acid and ethylene [33], and promote the synthesis of *PHR1* and *MYB62*, members of the MYB transcription factor family. They inhibit gibberellin synthesis and its signaling pathway [34, 35]. ABA-insensitive 5 protein (ABI5) binds to the embryonic specification element (ESE) of the *Dc3* gene promoter and ABA response element (ABRE). By controlling the expression of ABA regulatory genes during seed development, it directly affects ABA synthesis [36]. Moreover, *ERF7* can directly inhibit ABA synthesis by interacting with *SNL1* (SIN3-LIKE1) and *SNL2* in the presence of ethylene [37, 38]. By analyzing the interaction between mRNA and miRNA, we found that ath-miR167d binds to and inhibits ABI, limiting its synthesis. However, our relative expression levels analysis demonstrated that ath-miR167d expression level is low during apomixis, so it could not restrict ABA synthesis at this stage. Furthermore, miRNAs play a role in the auxin regulatory pathway. *AVP1* encodes a proton pump that regulates the pH in plant vacuoles. *AVP1* and *AUX1*/*IAA* participate in gradient regulation and transport of auxin [39, 40]. The expression of *AVP1* is restricted by ath-miR858a, and the relative expression level of the two is negatively correlated, as was shown by RT-qPCR analysis. An increase in ath-miR858a expression level in the ZB apomixis process inhibits the function of *AVP1*. In summary, we suggest that miRNA and its target genes participate in apomixis by regulating hormone synthesis.

## Conclusions

We have demonstrated through cytological observation that ZB employs the sporophyte apomixis type and that the S3 stage is a critical period in the process. We performed miRNA sequencing and RT-qPCR to screen candidate miRNAs involved in apomixis. Our results showed that miR172 had a higher expression level during the apomixis S3 period. Furthermore, the dual-luciferase reporter assay verified that miR172 regulates *TOE3* and *AP2* expression levels. These interact with *AGAMOUS* to regulate the apomixis process. This study showed evidence for the apomixis process in ZB and provided insights into its mechanism. It is necessary to clarify the roles of the regulatory factors at multiple levels and by multiple means to understand the apomixis mechanism more clearly, and, ultimately, describe its regulatory interaction network.

## Methods

### Plant materials

Plant materials were collected from the ZB Experimental Station, Northwest Agriculture and Forestry University, Fengxian, Shaanxi, China. Healthy, five-year-old ZB (*Zanthoxylum bungeanum* cv. Hanchengdahongpao) shrubs with uniform growth were selected. ZB is a dioecious plant. Unopened ZB female flowers were bagged and collected at four stages of development: S1, pre-flowering, flowers not to open; S2, mid-flowering, 7 days after flowering starts; S3, young fruit, 15 days after flowering starts; S4, fruit expansion, 30 days after flowering starts. Six-hundred flowers were collected from three ZB trees at each stage. The samples were immediately stored in liquid nitrogen for genetic analysis. Three biological replicates (three ZB trees) were collected at each developmental stage. The flowers or fruits used for cytological observations were stored in formalin-acetic acid-alcohol (FAA) fixative.

### miRNAs library construction and sequencing

Plant samples were sent to the Beijing Novogene Genomics Institute for miRNAs sequencing. The HiSeq and MiSeq techniques were used for miRNAs sequencing. The Small RNA Sample Preparation Kit (Illumina, San Diego, CA, USA) was used to construct sequencing libraries. The 12 miRNAs libraries established included three biological replicates at each of the four fruit development stages. The sRNA terminals were directly added with adaptors and reverse-transcribed to synthesize cDNA. PAGE gel electrophoresis was used to separate the target DNA fragments after PCR amplification. The cDNA libraries were obtained by cutting the gel and recovering the DNA. The adaptors and low-quality reads were removed from the raw reads to obtain the clean reads. Clean reads with a length of 18–30 nucleotides were kept for downstream analysis. The clean reads were compared with the sequence in miRBase, and the secondary structures of known miRNA, miRNA sequence, length, abundance, and other information were obtained. miREvo [41] and miRDeep2 [42] were used to predict and analyze novel miRNA. We then predicted the target genes of differentially expressed miRNAs and performed GO and KEGG enrichment analyses on these target genes.

### Differentially expressed miRNA analysis

Transcripts per million reads [TPM = (Read count*1,000,000)/ Mapped Reads count] represent the expression level of miRNAs. The DESeq software was used to analyze differentially expressed miRNAs between developmental stages. The screening criteria used were False Discovery Rate (FDR) ≤ 0.05 and │log2 FC│ ≥ 1, where FC is fold change.

### Total RNA extraction and cDNA synthesis

The TaKaRa MiniBEST Plant RNA Extraction Kit (TaKaRa, Beijing, China) was used to extract total RNA from the flowers or fruits at the different developmental stages. The first-strand cDNA synthesis for the miRNA was carried out using the Mir-X miRNA First-Strand Synthesis Kit (TaKaRa) according to the manufacturer’s instructions. These were used as templates for the RT-qPCR reactions.

### Quantitative real-time PCR (RT-qPCR)

The CFX96 Real-Time PCR Detection System (Bio-Rad, Hercules, CA, USA) was used to confirm the relative expression of miRNAs during apomixis. A reaction system of 10 μL contained 5 μL of 2× SYBR Premix Ex Taq II (TaKaRa), 1 μL of cDNA, 1 μL of each of the forward and universal reverse primers, and 2 μL of ddH_2_O. *U6* was used as an endogenous reference gene. Similarly, the relative expression levels of the target genes were detected by RT-qPCR, with *ZBUBQ* and *ZBTIF* as reference genes [43]. Primers were designed using Primer Premier 5.0 (Premier, Palo Alto, CA, USA). The primer sequences are shown in Table S3. The RT-qPCR reaction protocol was: 95 °C for 30 s followed by 40 cycles of 94 °C for 5 s, 54 °C for 30 s, and 72 °C for 45 s. Three technical replicates were made for each sample.

### Prediction of miRNAs target genes

The psRNATarget online tool (http://plantgrn.noble.org/psRNATarget/) uses the full-length transcriptome as the target template to predict the target genes of known and novel miRNAs. The specific parameters were set as follows: expectation: 5, penalty for G:U pair: 0.5, penalty for other mismatches: 1, extra weight in seed region: 1.5, seed region: 2–13 nucleotides, mismatches allowed in seed region: 2, HSP size: 19, penalty for opening gap: 2, penalty for extending gap: 0.5, and translation inhibition range: 10–11 nucleotides.

### Dual-luciferase reporter assay system

The dual-luciferase reporter assay system was used to verify the interaction between the miRNA and its target genes. The miRNA precursors and their target genes were constructed on pCA1301 and pBI121 vectors, respectively. A GUS (β-glucuronidase) reporter gene was inserted downstream of the pBI121 vector. If the miRNA cleaves the mRNA, the downstream GUS gene cannot be expressed, and thus the GUS protein cannot be detected. The interaction between miRNAs and target genes can thus be verified by detecting GUS protein expression [18].

The GUS staining experiment was carried out on four-week-old tobacco plant leaves (*Nicotiana benthamiana*). We detected the GUS activity in leaves injected with LB, 35S::*AP2*-GUS, 35S::miR172c + 35S::GUS, 35S::*AP2*-GUS + 35S::miR172c, 35S::*TOE3*-GUS, and 35S::*TOE3*-GUS + 35S::miR172c. We thoroughly grounded 0.5 g of the liquid nitrogen-frozen tobacco leaves, which were used as experimental material. We then used the GUS histochemical staining Kit (MaoKang, Shanghai, China), following the manufacturer’s instructions, to detect the GUS protein expression in them.

## Supplementary Information

**Additional file 1.**

## Data Availability

All the data and materials that are required to reproduce these findings can be shared by contacting the corresponding author, Prof. Anzhi Wei (weianzhi@126.com).

## Abbreviations

ABA: Abscisic acid
*ABI5*: *ABA-Insensitive 5 protein*
ABRE: ABA response element
*AP2*: *APETALA* 2
ARF: Auxin response factor
*BbGID*1: *Gibberellin-insensitive dwarf protein 1*
*BBM1*: *Baby boom 1*
ESE: Embryonic specification element
GO: Gene Ontology
KEGG: Kyoto Encyclopedia of Genes and Genomes
lncRNA: Long non-coding RNA
PCA: Principal component analysis
sRNA: small RNA
TPM: Transcripts per million reads
ZB: *Zanthoxylum bungeanum*
GUS: β-glucuronidase
SNL1: SIN3-LIKE1
