# Supplementary material for: Small RNA sequencing provides candidate miRNA-target pairs for revealing the mechanism of apomixis in Zanthoxylum bungeanum
Source: BMC Plant Biol. 2021 Apr 13;21:178. doi: 10.1186/s12870-021-02935-5 (PMC8042946; doi:10.1186/s12870-021-02935-5)
Supplement: Supplementary file 1 — Additional file 1. [file 12870_2021_2935_MOESM1_ESM.docx]

### miRNA sequencing reveals the dynamic regulation of miRNA-target pairs during apomixis in *Zanthoxylum bungeanum*

Xitong Fei^1,2,†^, Yu Lei^1,2,†^, Yichen Qi^1,2^, Shujie Wang^1,2^, Haichao Hu^1,2^, Anzhi Wei^1,2,^*

^1^ College of Forestry, Northwest Agriculture and Forestry University, Xianyang 712100, China

^2^ Research Centre for Engineering and Technology of Zanthoxylum State Forestry Administration, Yangling, Xianyang 712100, China

† These authors contributed equally to this work.

*Corresponding author. E-mail address: weianzhi@126.com

**Supplemental material**

**Figure S1. Kyoto Encyclopedia of Genes and Genomes (KEGG) enrichment analysis of the differentially expressed miRNAs target genes.**

**
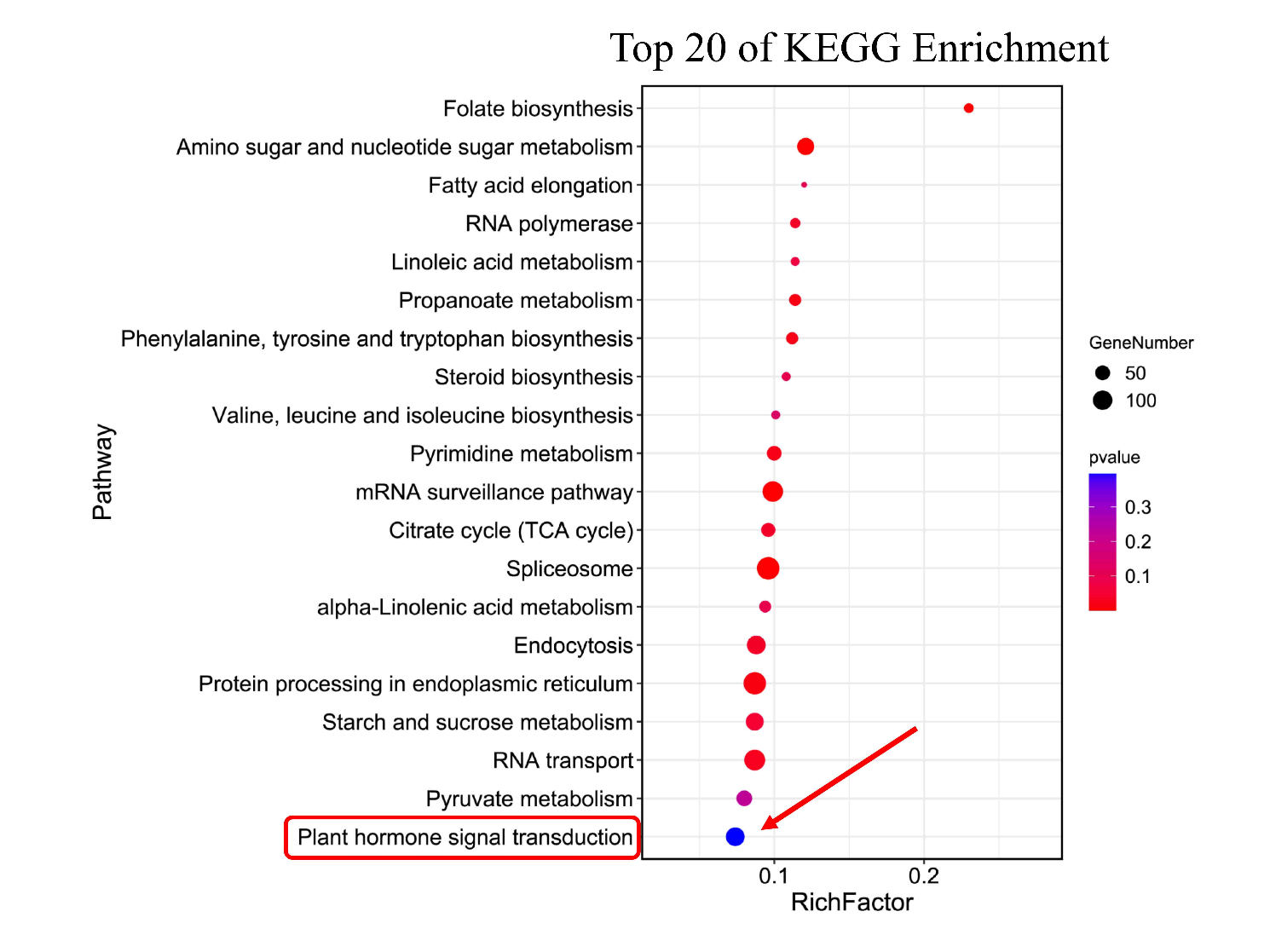
**

**Table S1. Quantitative distribution of miRNAs in the four fruit developmental stages in *Zanthoxylum bungeanum.***

| **Types** | **Total** | **S1** | **S2** | **S3** | **S4** |
| --- | --- | --- | --- | --- | --- |
| known miRNA mature | 40 | 34 | 33 | 31 | 30 |
| known miRNA hairpins | 49 | 41 | 45 | 44 | 38 |
| novel miRNA mature | 56 | 46 | 35 | 51 | 34 |
| novel miRNA hairpins | 57 | 52 | 43 | 54 | 38 |

**Table S2. miRNAs and their target genes.**

| **miRNA** | **Mature miRNA sequence** | **Target gene description** |
| --- | --- | --- |
| miR172c | AGAAUCUUGAUGAUGCUGCAG | Transcription factor TOE3 |
| miR172c | AGAAUCUUGAUGAUGCUGCAG | Transcription factor RAP2-7 |
| miR172c | AGAAUCUUGAUGAUGCUGCAG | Transcription factor RAP2-7-like (LOC109342033) |
| miR172c | AGAAUCUUGAUGAUGCUGCAG | Floral homeotic protein APETALA 2 |
| miR172c | AGAAUCUUGAUGAUGCUGCAG | Polyadenylate-binding protein RBP47 |
| miR167d | UGAAGCUGCCAGCAUGAUCUGG | alpha-trehalose-phosphate synthase |
| miR167d | UGAAGCUGCCAGCAUGAUCUGG | Histone-lysine N-methyltransferase TRX1 |
| miR167d | UGAAGCUGCCAGCAUGAUCUGG | Disulfide-isomerase |
| miR167d | UGAAGCUGCCAGCAUGAUCUGG | heterogeneous nuclear ribonucleoprotein A3 |
| miR167d | UGAAGCUGCCAGCAUGAUCUGG | Metal transporter Nramp1 |
| ath-miR319a | UUGGACUGAAGGGAGCUCCCU | Transcription factor GAMYB |
| ath-miR319a | UUGGACUGAAGGGAGCUCCCU | Carbamoyl-phosphate synthase arginine-specific large chain |
| ath-miR319a | UUGGACUGAAGGGAGCUCCCU | Transcription factor TCP2 |
| ath-miR160a-5p | UGCCUGGCUCCCUGUAUGCCA | Auxin response factor 16 |
| ath-miR160a-5p | UGCCUGGCUCCCUGUAUGCCA | Auxin response factor 18 |
| ath-miR160a-5p | UGCCUGGCUCCCUGUAUGCCA | Auxin response factor 17 |
| ath-miR160a-5p | UGCCUGGCUCCCUGUAUGCCA | Mitogen-activated protein kinase kinase kinase 15 |
| ath-miR160a-5p | UGCCUGGCUCCCUGUAUGCCA | Mitogen-activated protein kinase kinase kinase 5 |
| ath-miR160a-5p | UGCCUGGCUCCCUGUAUGCCA | Probable serine/threonine-protein kinase fhkC |
| ath-miR858a | UUUCGUUGUCUGUUCGACCUU | Type I proton-pumping pyrophosphatas |
| ath-miR858a | UUUCGUUGUCUGUUCGACCUU | Transcription factor MYB3 |
| ath-miR858a | UUUCGUUGUCUGUUCGACCUU | CWF19-like protein 2 |
| ath-miR858a | UUUCGUUGUCUGUUCGACCUU | Transcription repressor MYB6-like |
| ath-miR858a | UUUCGUUGUCUGUUCGACCUU | E3 ubiquitin-protein ligase SDIR1 |
| ath-miR858a | UUUCGUUGUCUGUUCGACCUU | Transcription factor MYB4 |
| ath-miR858a | UUUCGUUGUCUGUUCGACCUU | Transcription factor MYB24 |
| ath-miR858a | UUUCGUUGUCUGUUCGACCUU | Transcription factor MYB35 |
| ath-miR858a | UUUCGUUGUCUGUUCGACCUU | Lysine-specific demethylase JMJ25 |
| ath-miR166a-5p | GGACUGUUGUCUGGCUCGAGG | Transcription factor WRKY75 |
| ath-miR166a-5p | GGACUGUUGUCUGGCUCGAGG | DUF724 domain-containing protein 3 |
| ath-miR166a-5p | GGACUGUUGUCUGGCUCGAGG | Nardilysin-like |
| ath-miR166a-5p | GGACUGUUGUCUGGCUCGAGG | Disease resistance protein RPP13-like |
| ath-miR166a-5p | GGACUGUUGUCUGGCUCGAGG | Protein-L-isoaspartate O-methyltransferase 1 |
| ath-miR166a-5p | GGACUGUUGUCUGGCUCGAGG | Dynamin-2A-like |
| ath-miR166a-5p | GGACUGUUGUCUGGCUCGAGG | Nardilysin |
| ath-miR166a-5p | GGACUGUUGUCUGGCUCGAGG | Auxilin-like protein 1 |
| ath-miR166a-5p | GGACUGUUGUCUGGCUCGAGG | Uncharacterized secreted glycosidase ARB_07629 |
| ath-miR162a-3p | GGACUGUUGUCUGGCUCGAGG | Endoribonuclease Dicer homolog 1 |
| ath-miR162a-3p | GGACUGUUGUCUGGCUCGAGG | Ca2+-binding transmembrane protein LETM1/MRS7 |
| ath-miR162a-3p | GGACUGUUGUCUGGCUCGAGG | Protein trichome birefringence-like 8 |
| ath-miR162a-3p | GGACUGUUGUCUGGCUCGAGG | LETM1 and EF-hand domain-containing protein |
| ath-miR162a-3p | GGACUGUUGUCUGGCUCGAGG | Putative UDP-sugar transporter DDB_G0278631 |
| ath-miR162a-3p | GGACUGUUGUCUGGCUCGAGG | Heat shock 70 kDa protein 16 |
| ath-miR162a-3p | GGACUGUUGUCUGGCUCGAGG | Uncharacterized LOC102625692 |
| ath-miR162a-3p | GGACUGUUGUCUGGCUCGAGG | Disease resistance protein At1g61190 |
| ath-miR157d | UGACAGAAGAUAGAGAGCAC | Abscisic acid-insensitive 5 |
| ath-miR157d | UGACAGAAGAUAGAGAGCAC | Squamosa promoter-binding-like protein 12 |
| ath-miR157d | UGACAGAAGAUAGAGAGCAC | Squamosa promoter-binding-like protein 12 (LOC102610993) |
| ath-miR157d | UGACAGAAGAUAGAGAGCAC | Squamosa promoter-binding-like protein 2 |
| ath-miR157d | UGACAGAAGAUAGAGAGCAC | Aquamosa promoter-binding-like protein 13A |
| ath-miR157d | UGACAGAAGAUAGAGAGCAC | Squamosa promoter-binding-like protein 6 |
| ath-miR156b-3p | UGCUCACCUCUCUUUCUGUCAGU | DEAD-box ATP-dependent RNA helicase 20 (LOC102619005) |
| ath-miR156b-3p | UGCUCACCUCUCUUUCUGUCAGU | LOW QUALITY PROTEIN LOC102610230 |
| ath-miR156b-3p | UGCUCACCUCUCUUUCUGUCAGU | Elongation factor Ts |
| ath-miR156b-3p | UGCUCACCUCUCUUUCUGUCAGU | DNA mismatch repair protein MLH1 |
| ath-miR156b-3p | UGCUCACCUCUCUUUCUGUCAGU | Transcription factor BIM3 |
| ath-miR156b-3p | UGCUCACCUCUCUUUCUGUCAGU | Serine/threonine-protein phosphatase PP1 |
| ath-miR156b-3p | UGCUCACCUCUCUUUCUGUCAGU | Bifunctional dihydrofolate reductase-thymidylate synthase |

**Table S3.Primers for real-time quantitative PCR.**

| **Name** | **Description** | **Forward** | **Reverse** | **Product Length** |
| --- | --- | --- | --- | --- |
| ***WRKY75*** | WRKY transcription factor 75 | AGTGGGTTCTTGGGATTATTG | GATGCTGGTGCTACTGCTGTT | 99 |
| ***ABI5*** | ABSCISIC ACID-INSENSITIVE 5-like protein | AGGATCTTTAACTTTGCCACG | CACTCACATCTTCAGCACCAG | 101 |
| ***AP2*** | APETALA 2 | ACATCCAATGCCACCAACAAT | CAACATCGAATCCAAGAGCGT | 195 |
| ***TOE3*** | Transcription factor TOE3 | GGAGTTTGCGTTTTTTCCTGT | TTGCTGCTTCTGCTGCTTATC | 91 |
| ***TCP2*** | Transcription factor TCP2 | TGGATTATTTCTACTCGGGGT | TGGTGGGATTGTCATTGCTTG | 119 |
| ***IAA26*** | Auxin-responsive protein IAA26 | TGATCTCAATGCTTATGACAG | AAACAAGAGTATATTCCCCAC | 173 |
| ***AVP*** | Vacuolar H+-PPase | CTGGAGGCTAGAAAGGGAGTG | TGAATAGGTTGATGGCAATGA | 121 |
| ***MYB3*** | Transcription factor MYB3 | CTCACAGATTACATCAAGGC | TCTCAAATAGTTCATCCAGC | 114 |
| ***ZbUBQ*** | Reference gene 1 | TCGAAGATGGCCGTACATTG | TCCTCTAAGCCTCAGCACCA | 122 |
| ***ZbTIF*** | Reference gene 2 | TTCCTCCCATTACGTTGCT | GCTGGTTACGGACTCTTTG | 165 |
